# Supplementary figures and images for: Neonatal valproic acid exposure produces altered gyrification related to increased parvalbumin-immunopositive neuron density with thickened sulcal floors
Source: PLoS One. 2021 Apr 20;16(4):e0250262. doi: 10.1371/journal.pone.0250262 (PMC8057614; doi:10.1371/journal.pone.0250262)

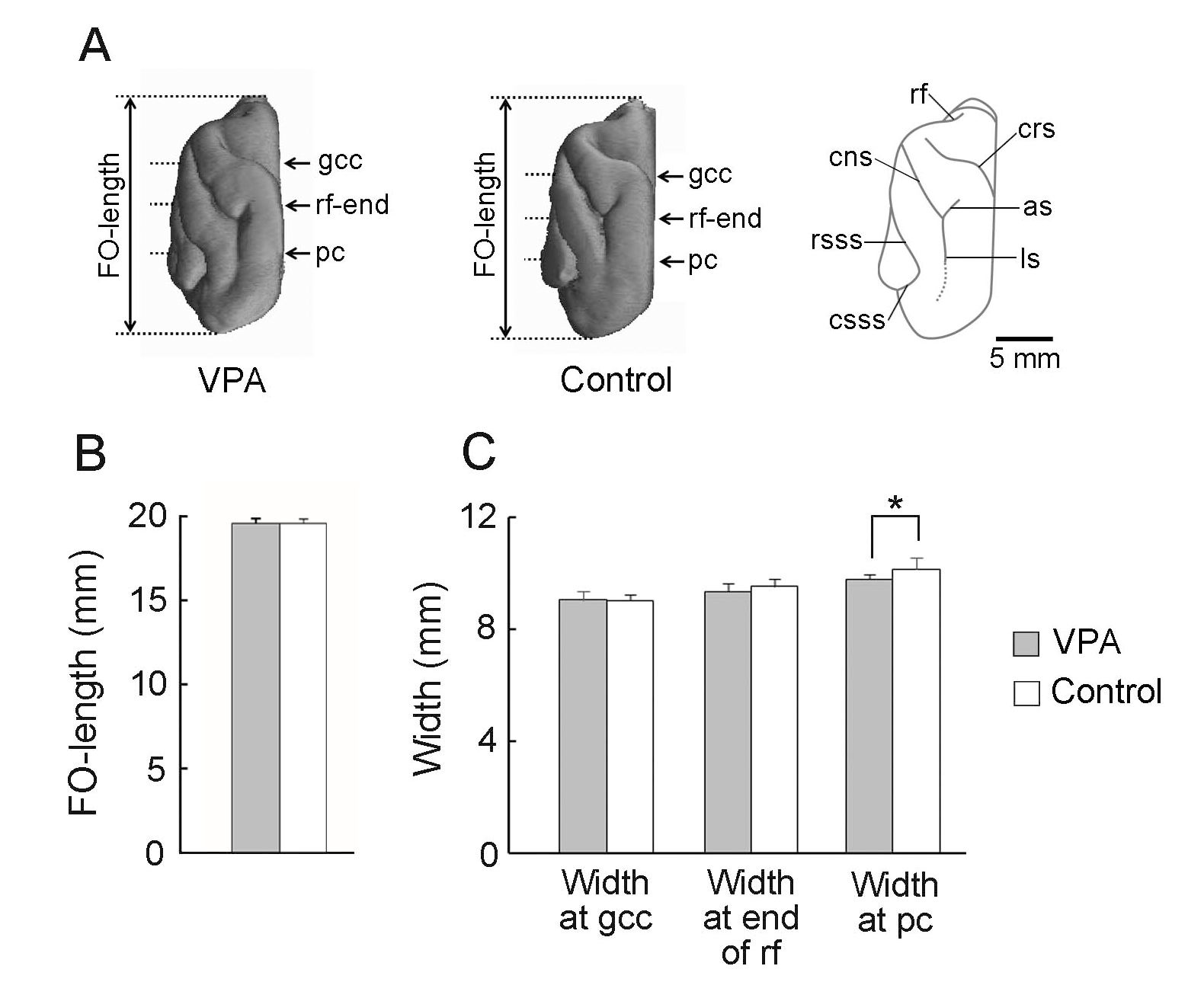

Supplement: S1 Fig — [A] 3D-rendered images of the left cerebral hemisphere, dorsal view, indicating measurement references for FO-length and the width of the cerebral hemisphere [VPA-treated, left; control treated, center]. Arrows indicate measurement points for the cerebral width at the genu of the corpus callosum [gcc], caudal end of the rhinal fissure [rf], and the posterior commissure [pc]. An illustration of the dorsal surface of the left hemisphere indicating the primary gyri and sulci is shown on the right. [B] FO-length of the cerebral hemisphere. [C] Width of the cerebral hemisphere. [D] Cortical volume. Data are shown as mean ± SEM. Significance is indicated using Scheffe’s test at * P < 0.001; number of cerebral hemispheres = 8. AEG, anterior ectosylvian gyrus; ASG, anterior sigmoid gyrus; CNG, coronal gyrus; cns, coronal sulcus; crs, cruciate sulcus; csss, caudal suprasylvian; LG, lateral gyrus; ls, lateral sulcus; PSG, posterior sigmoid gyrus; rsss, rostral suprasylvian sulcus; SSG, suprasylvian sulcus; VCA, visual cortical area. (TIF) [file pone.0250262.s001.tif]

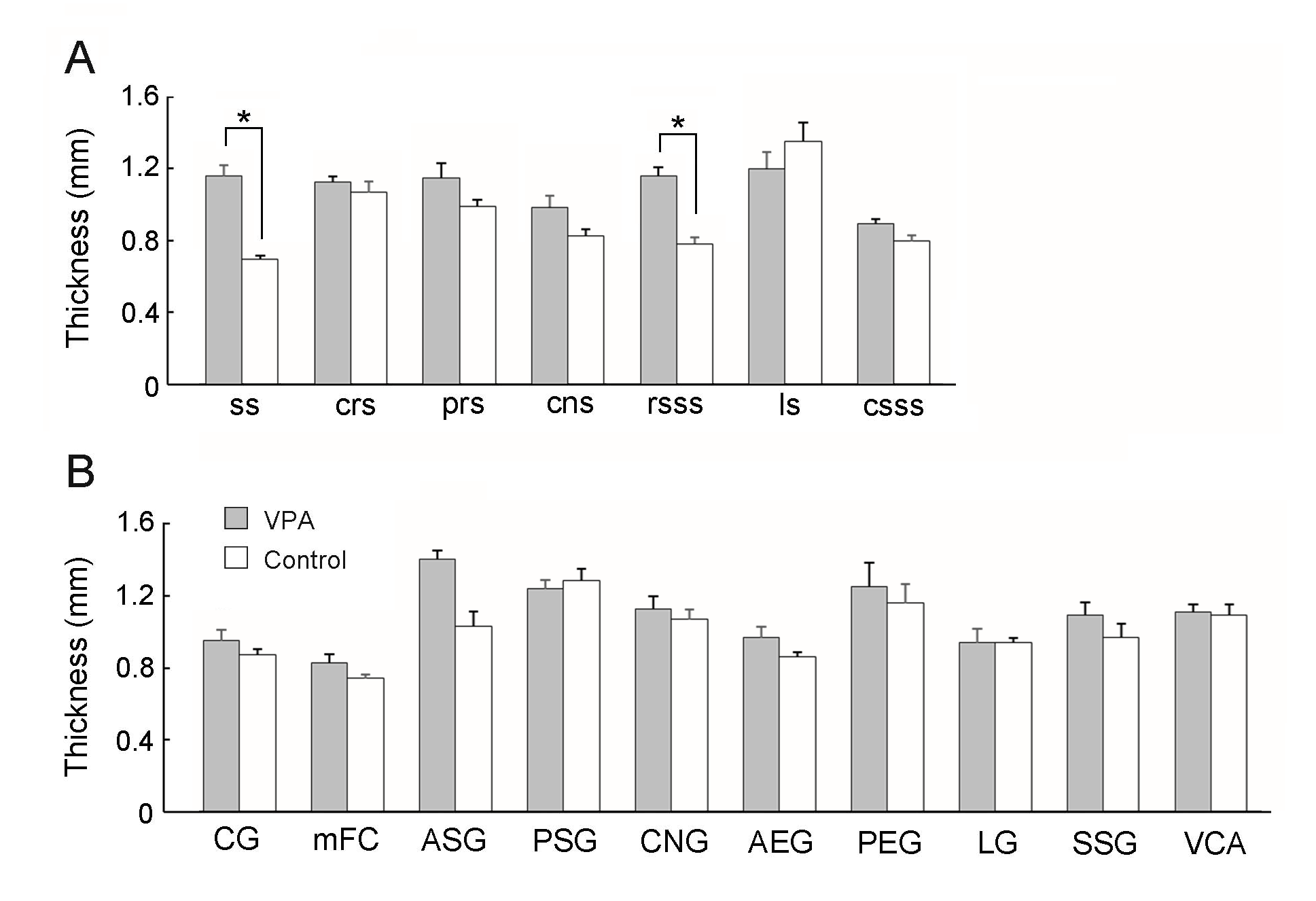

Supplement: S2 Fig — [A] Bar graphs displaying cortical thickness of the sulcal floors. [B] Cortical thickness of the gyral crowns. Data are shown as mean ± SEM. Significance is indicated using Scheffe’s test at * P < 0.001; number of cerebral hemispheres = 8. cns, coronal sulcus; crs, cruciate sulcus; csss, caudal suprasylvian; ls, lateral sulcus; prs, presylvian sulcus; rsss, rostral suprasylvian sulcus; ss, splenial sulcus; AEG, anterior ectosylvian gyrus; ASG, anterior sigmoid gyrus; CG, cingulate gyrus; CNG, coronal gyrus; mFC, medial frontal cortex; LG, lateral gyrus; PEG, posterior ectosylvian gyrus; PSG, posterior sigmoid gyrus; SSG, suprasylvian gyrus; VCA, visual cortical area. (TIF) [file pone.0250262.s002.tif]

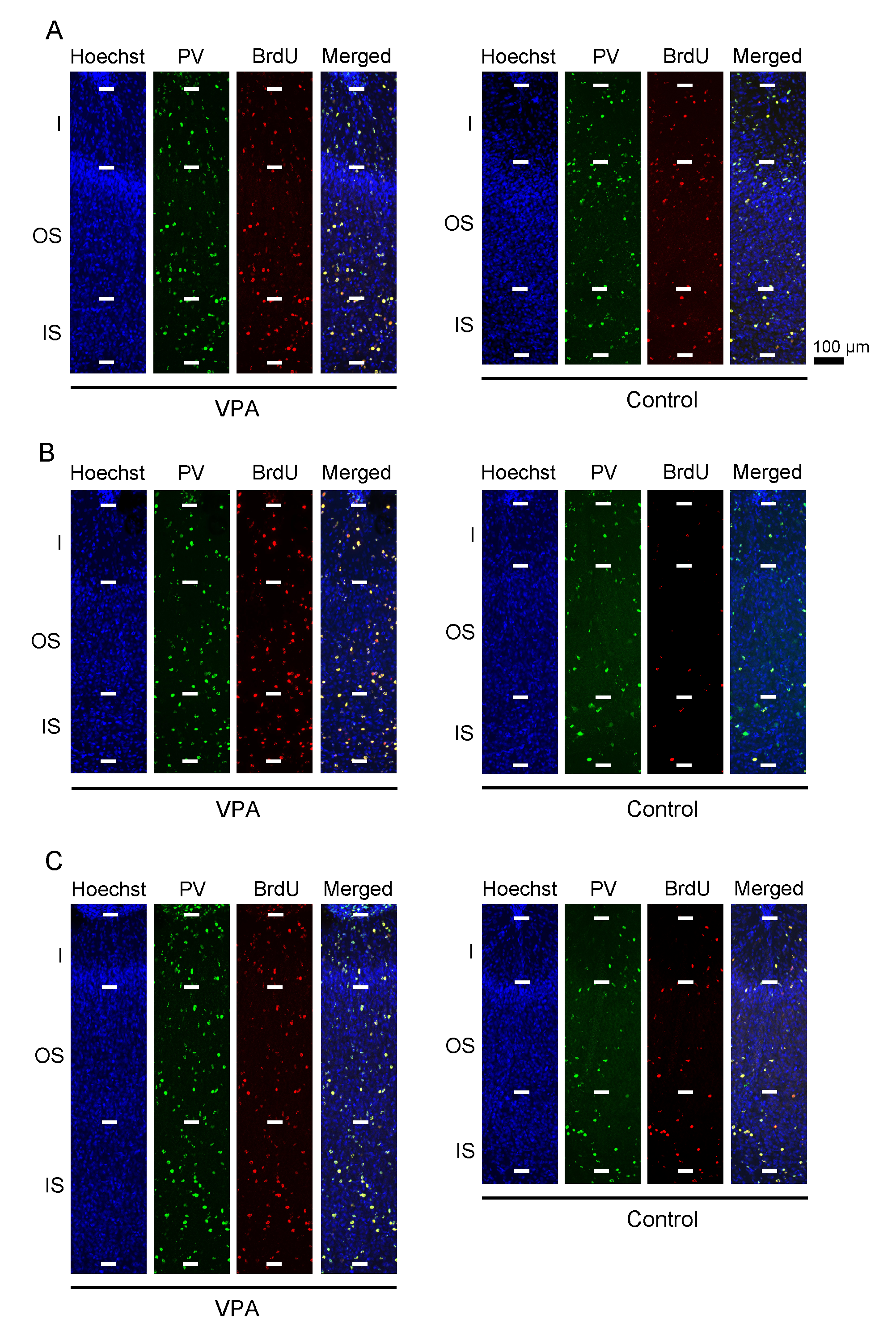

Supplement: S3 Fig — (A) Cortical depth of the presylvian sulcus (prs) floor. (B) Cortical depth of the coronal sulcus (cns) floor. (C) Cortical depth of the splenial sulcus (ss) floor. PV, parvalbumin. (TIF) [file pone.0250262.s003.tif]

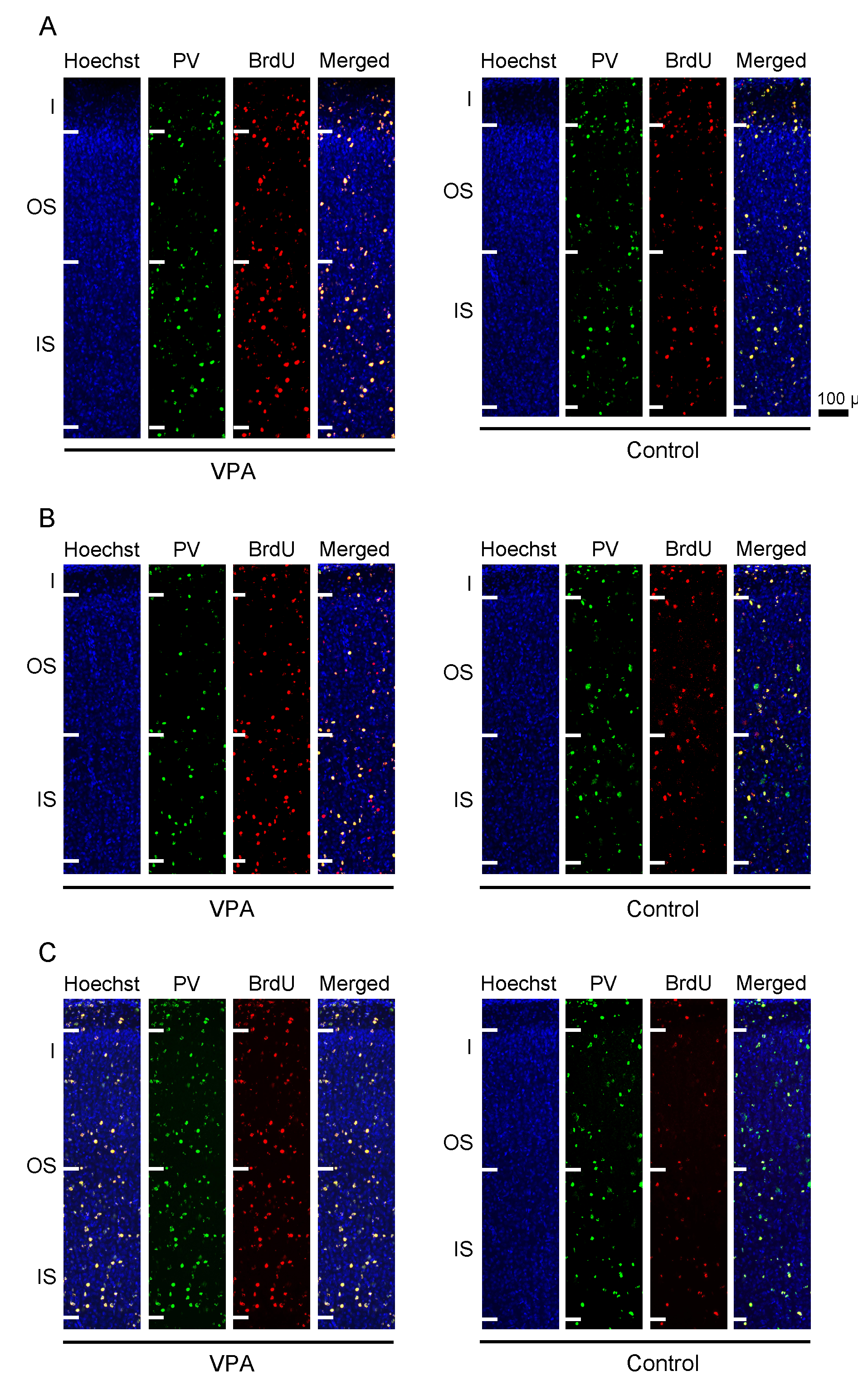

Supplement: S4 Fig — (A) Cortical depth of the anterior sigmoid gyrus (ASG) crown. (B) Cortical depth of the coronal gyus (CNG) crown. (C) Cortical depth of the suprasylvian gyrus (SSG) crown. PV, parvalbumin. (TIF) [file pone.0250262.s004.tif]

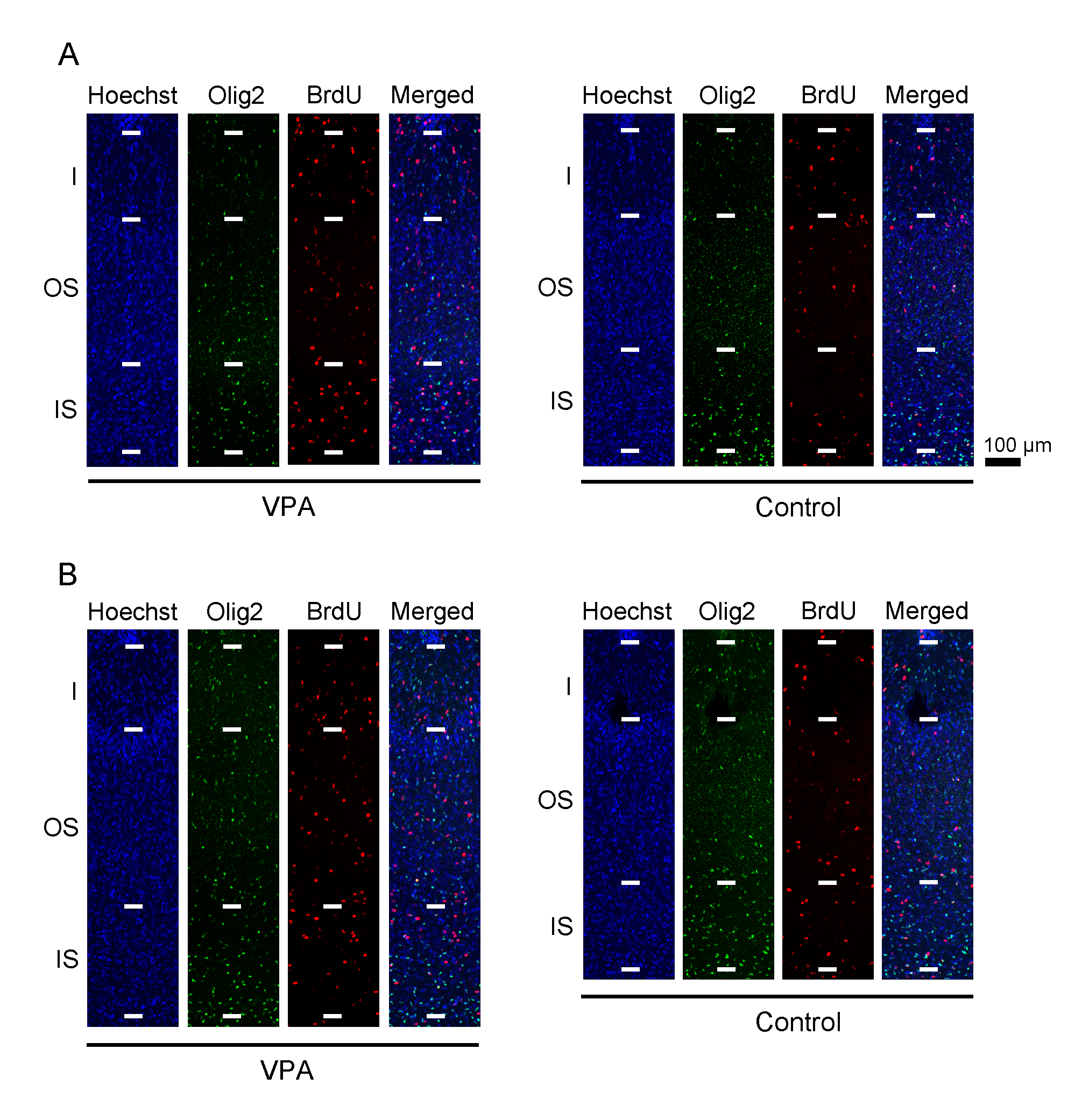

Supplement: S5 Fig — (A) Cortical depth of the rostral sylvian sulcus (rsss) floor. (B) Cortical depth of the coronal sulcus (cns) floor. (TIF) [file pone.0250262.s005.tif]
