# Supplementary material for: Neonatal valproic acid exposure produces altered gyrification related to increased parvalbumin-immunopositive neuron density with thickened sulcal floors
Source: PLoS One. 2021 Apr 20;16(4):e0250262. doi: 10.1371/journal.pone.0250262 (PMC8057614; doi:10.1371/journal.pone.0250262)
Supplement: S1 Table — (PDF) [file pone.0250262.s006.pdf]

S1 Table. Body and brain weights of PD 20 ferrets used in the present study.

	VPA (n=4)	Control (n=4)
Body weight (g)	102.6 ± 3.5*	75.8 ± 4.1
Brain weight (mg)	3,456 ± 133	3,275 ± 177

Data are represented as mean ± SEM. *$P < 0.001$ vs Control (Student's *t*-test)
