# Supplementary material for: Neonatal valproic acid exposure produces altered gyrification related to increased parvalbumin-immunopositive neuron density with thickened sulcal floors
Source: PLoS One. 2021 Apr 20;16(4):e0250262. doi: 10.1371/journal.pone.0250262 (PMC8057614; doi:10.1371/journal.pone.0250262)
Supplement: S2 Table — (PDF) [file pone.0250262.s007.pdf]

S2 Table. Percentage of PV-positive/BrdU-labeled cells in sulcal floors and gyral crowns in PD 20 ferrets.

	VPA		Control	
Sulcal floors				
Presylvian sulcus (prs)	73.9%	(34/49)	69.0%	(29/43)
Coronal sulcus (cns)	58.0%	(29/50)	72.2%	(26/36)
Rostral suprasylvian sulcus (rsss)	44.4%	(20/45)*	15.6%	(5/32)
Lateral sulcus (ls)	75.5%	(38/51)	57.9%	(22/38)
Splenial sulcus (ss)	50.7%	(34/67)	37.0%	(10/27)
Gyral crowns				
Anterior sigmoid gyrus (ASG)	54.7%	(47/86)	45.7%	(32/70)
Coronal gyrus (CNG)	49.1%	(28/57)	46.2%	(24/52)
Suprasylvian gyrus (SSG)	47.4%	(54/114)	55.8%	(29/52)

Percentages were calculated by summing each immunolabeled cell counted within all ROIs from eight cerebral hemispheres in each group. The number of each labeled cell for calculating the percentages is shown in parentheses as a function of total BrdU-labeled cells from eight cerebral hemispheres in each group. * $P < 0.01$ (χ square test)
