# Supplementary material for: Neonatal valproic acid exposure produces altered gyrification related to increased parvalbumin-immunopositive neuron density with thickened sulcal floors
Source: PLoS One. 2021 Apr 20;16(4):e0250262. doi: 10.1371/journal.pone.0250262 (PMC8057614; doi:10.1371/journal.pone.0250262)
Supplement: S3 Table — (PDF) [file pone.0250262.s008.pdf]

S3 Table. Percentage of Olig2-positive/BrdU-labeled cells in the coronal and rostral suprasylvian sulci floors in PD 20 ferrets.

	VPA		Control	
Sulcal floors				
Coronal sulcus (cns)	25.0%	(12/48)	17.4%	(4/23)
Rostral suprasylvian sulcus (rsss)	27.1%	(13/48)	19.2%	(5/26)

Percentages were calculated by summing each immunolabeled cell counted within all ROIs from eight cerebral hemispheres in each group. The number of each labeled cell for calculating the percentages is shown in parentheses as a function of total BrdU-labeled cells from eight cerebral hemispheres in each group.
