# Supplementary material for: Neonatal valproic acid exposure produces altered gyrification related to increased parvalbumin-immunopositive neuron density with thickened sulcal floors
Source: PLoS One. 2021 Apr 20;16(4):e0250262. doi: 10.1371/journal.pone.0250262 (PMC8057614; doi:10.1371/journal.pone.0250262)
Supplement: S4 Table — (PDF) [file pone.0250262.s009.pdf]

S4 Table . Olig2-positive cell densities with or without BrdU labeling in the coronal and rostral suprasylvian sulci floors of PD 20 ferrets.

	n=	IS		OS	
		cns	rsss	cns	rsss
Olig2+ cells (/mm^3)					
VPA	8	65,104 ± 7,634	52,083 ± 8,612	94,401 ± 9,967	91,146 ± 7,634
Control	8	69,987 ± 7,953	60,221 ± 9,473	79,753 ± 12,489	86,263 ± 12,382
Olig2+/BrdU+ cells (/mm^3)					
VPA	8	6.510 ± 3,255	6,510 ± 2,302	13,021 ± 2,302	14,648 ± 4,268
Control	8	3,255 ± 3,045	1,628 ± 1,522	3,255 ± 1,993	6,510 ± 3,255

Density data are represented as mean ± SEM of eight cerebral hemispheres.
"+" indicates "immunopositive."
